# Extracellular vesicle-mediated bidirectional communication between the brain and peripheral organs in Alzheimer’s disease: evidence, mechanisms, and translational perspectives

**DOI:** 10.1186/s12951-026-04705-7

**Published:** 2026-06-26

**Authors:** Yuqing Liu, Zhongliu Yao, Lemei Zhu, Weijun Peng

**Affiliations:** 1 Department of Integrated Traditional Chinese & Western Medicine, The Second Xiangya Hospital, Central South University, No.139 Middle Renmin Road, Changsha, 410011 Hunan People’s Republic of China; 2 National Clinical Research Center for Metabolic Diseases, The Second Xiangya Hospital, Central South University, Changsha, China; 3 Department of Cardiology, Third Xiangya Hospital of Central South University, Changsha, China; 4 Academician Workstation, Changsha Medical University, Changsha, China

**Keywords:** Extracellular vesicles, Alzheimer’s disease, Interorgan communication, Biomarkers, Therapeutic targets

## Abstract

Alzheimer’s disease (AD) has traditionally been conceptualized as a brain-centered neurodegenerative disorder characterized by amyloid-β (Aβ) deposition, tau pathology, synaptic dysfunction, and progressive neuronal loss. However, accumulating evidence suggests that AD is also shaped by systemic disturbances and reciprocal communication between the central nervous system and peripheral organs. Extracellular vesicles (EVs), which transport proteins, lipids, metabolites, and nucleic acids across biological fluids and barriers, have emerged as plausible mediators of this inter-organ crosstalk. In this Review, current evidence for EV-mediated bidirectional communication between the brain and peripheral organs is synthesized, with particular attention to the liver–brain, heart–brain, gut–brain, lung–brain, bone–brain, and adipose–brain axes. The strength of evidence across these axes is compared, and the ways in which organ-derived EVs may influence neuroinflammation, neurovascular dysfunction, metabolic homeostasis, blood–brain barrier integrity, and Aβ/tau-related processes are discussed, while also considering how brain-derived EVs (BDEVs) may affect peripheral physiology. The translational potential of EVs as diagnostic biomarkers, therapeutic carriers, and candidate targets for systemic intervention in AD is further evaluated. Current evidence most strongly supports the gut–brain, liver–brain, and adipose–brain axes, whereas several other axes remain supported primarily by experimental models or engineered EV studies. Major barriers to progress include EV heterogeneity, limited source specificity, insufficient standardization of isolation and quantification workflows, and a continuing reliance on associative human data and preclinical models. Overall, EVs are best viewed as one candidate signaling layer within a broader systemic network linking peripheral physiology to brain pathology. Clarifying the magnitude, directionality, and causal significance of these interactions will require rigorous EV characterization, source-resolved in vivo trafficking studies, and longitudinal clinical investigation.

**Graphical abstract:**

**Figure:**
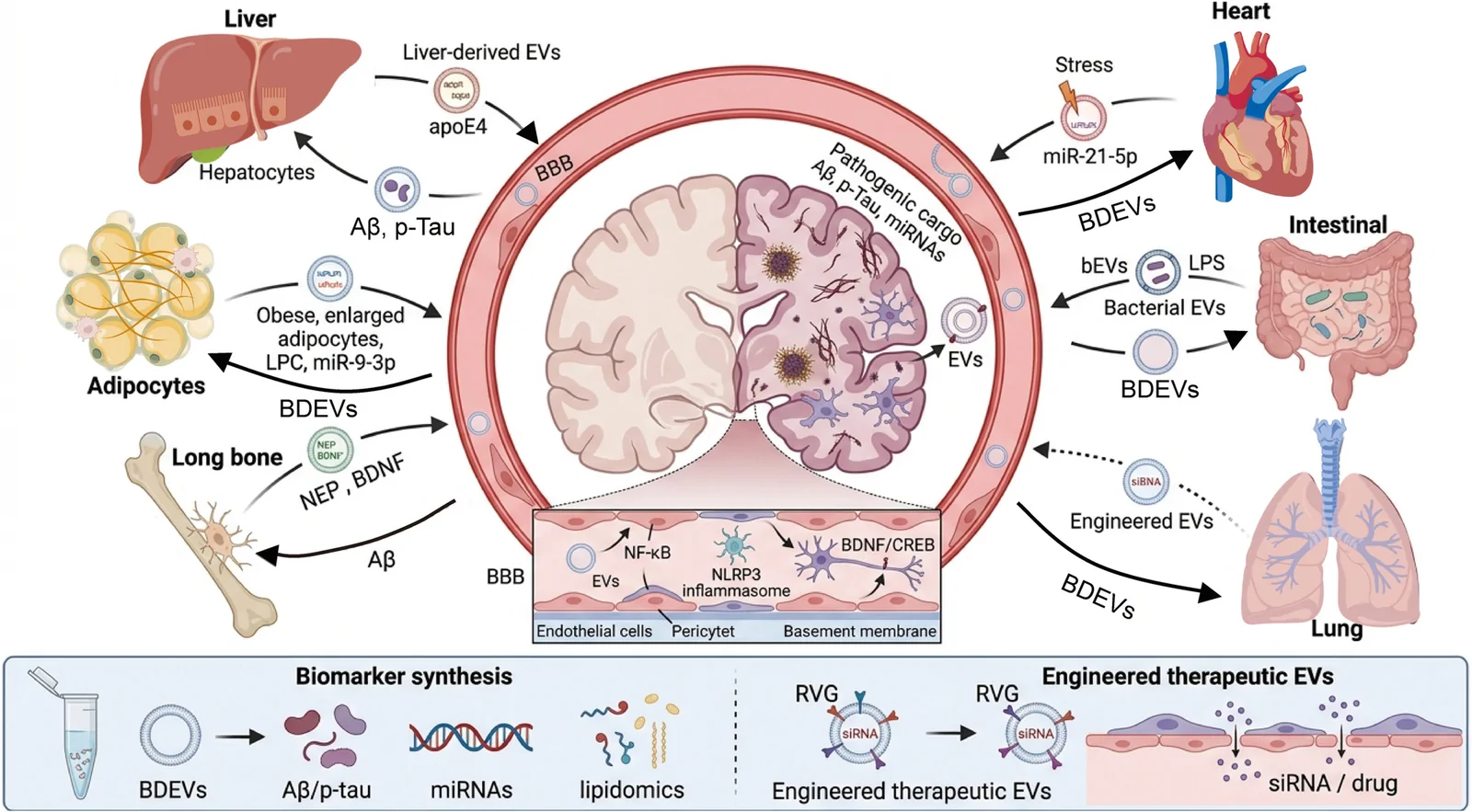

## Highlights

1. Extracellular vesicles (EVs) are plausible mediators of bidirectional communication between the brain and peripheral organs in Alzheimer’s disease (AD).
2. The gut–brain, liver–brain, and adipose–brain axes currently show the strongest convergent support for EV-related involvement in AD-relevant pathology.
3. Evidence for several other organ axes remains more model-dependent and is often confounded by limited source attribution or reliance on engineered EV systems.
4. EVs hold promise as biomarkers and therapeutic carriers, but translation is constrained by heterogeneity, uncertain organ-of-origin assignment, and limited methodological standardization.
5. Future progress will depend on source-resolved in vivo tracing, EV-specific causal perturbation, and longitudinal clinically relevant study designs.

## Introduction

Alzheimer’s disease (AD) is one of the most pressing health challenges facing ageing societies. Dementia as a whole affects more than 55 million people worldwide, with AD accounting for an estimated 60–70% of cases, imposing a rapidly increasing social and economic burden [1–3]. For decades, AD has been studied primarily through a brain-centered framework focused on amyloid-β (Aβ) plaques, neurofibrillary tangles composed of hyperphosphorylated tau, synaptic dysfunction, and progressive neuronal loss [4, 5]. This framework has substantially advanced understanding of neurodegenerative mechanisms and supported the development of symptomatic and, more recently, biomarker-informed therapeutic approaches. Nevertheless, the clinical impact of many brain-targeted interventions remains limited, and durable disease modification has proved difficult to achieve [6]. These limitations suggest that a strictly brain-confined model may be insufficient to explain the full biological complexity of AD.

A growing body of evidence suggests that AD is influenced by systemic physiology and by bidirectional interactions between the central nervous system and peripheral organs [7–9]. Epidemiological studies associate peripheral organ dysfunction-including hepatic steatosis, cardiovascular disease, chronic kidney disease, and metabolic syndrome with elevated AD risk, often years before overt cognitive symptoms [10, 11]. Mechanistic studies further indicate that peripheral inflammation, metabolic dysregulation, and vascular alterations can perturb brain homeostasis through impaired clearance of neurotoxic proteins, chronic low-grade inflammation, oxidative stress, and altered immune signaling [12, 13]. In addition, clinical and interventional observations suggest that improving peripheral health, particularly cardiovascular and metabolic status, may modify cognitive trajectories in some risk populations [10]. Together, these findings support the view that peripheral organs may shape AD susceptibility and progression, although the hierarchy, directionality, and molecular mediators of organ–brain communication remain incompletely defined.

Inter-organ communication is mediated by multiple classes of signals, including hormones, cytokines, metabolites, and extracellular vesicles (EVs) [14]. Among these, EVs have attracted particular interest because they are membrane-enclosed particles capable of protecting and transporting proteins, lipids, and nucleic acids through biological fluids. This makes them plausible candidates for mediating organ–brain crosstalk in AD. Brain-derived EVs (BDEVs) have been reported to enter systemic circulation and interact with peripheral tissues, raising the possibility that they contribute to CNS-to-periphery signaling [15]. Under stress or neurodegenerative conditions, neuronal and glial EVs may carry disease-relevant cargos, including Aβ-related species, α-synuclein, and regulatory microRNAs [16]. Recent proteomic studies further suggest that EV composition and surface interaction networks can be altered in AD-related models [17]. However, the functional significance, endogenous biodistribution, and in vivo specificity of these disease-associated EV signatures remain incompletely resolved. Accordingly, EVs should currently be regarded as plausible mediators rather than established dominant drivers of systemic pathological propagation in AD [18].

Conversely, EVs secreted by peripheral organs including adipose tissue, liver, gut, lung, bone, and heart, have been proposed to influence the brain through circulatory transport and cargo-dependent signaling [19–21]. For example, adipose-derived EVs have been linked to inflammatory and metabolic signaling relevant to hypothalamic dysfunction and neuroinflammation in obesity-related contexts [22, 23]. Liver-derived EVs have been implicated in blood–brain barrier modulation and neuroimmune regulation under conditions of metabolic stress, whereas microbiota- and gut-derived EVs have been proposed to influence neuroimmune responses through pattern recognition signaling and microbe-host metabolic interactions [24]. Taken together, these findings suggest that EVs may represent a dynamic signaling layer within a broader inter-organ network that links peripheral physiology to brain vulnerability. At the same time, major unresolved questions remain regarding cell-type-specific EV tracing, endogenous biodistribution, target-cell engagement, and the causal contribution of specific cargos.

In this Review, EV-mediated communication across major brain–periphery axes relevant to AD is examined, with emphasis on the liver, heart, gut, lung, bone, and adipose tissue. Rather than treating all axes as equally established, their relative evidentiary maturity is compared, endogenous pathogenic EV signaling is distinguished from engineered therapeutic EV applications, and the key methodological bottlenecks that currently limit causal inference and clinical translation are identified. By situating AD within an inter-organ communication framework, this Review clarifies where evidence is strongest, where major uncertainties remain, and which next-step study designs are most likely to advance the field.

### Literature search approach for this narrative review

To support this narrative review, we conducted a structured literature search using PubMed, Web of Science, Scopus, and Embase. The search covered publications from January 2000 to May 27, 2026 (the final search update date) and was designed to capture foundational studies and recent advances in extracellular vesicle biology, Alzheimer’s disease, organ–brain communication, and translational applications.

Search terms combined concepts related to extracellular vesicles, exosomes, microvesicles, Alzheimer’s disease, neurodegeneration, organ systems, biomarkers, and therapy using Boolean operators. Additional terms included “brain-derived EVs”, “BDEVs”, “organ–brain axis”, “blood–brain barrier”, “Aβ”, “tau”, “miRNA”, “neuroinflammation”, “engineered EVs”, and “clinical translation”. Searches were iteratively refined according to organ axis and translational theme, and reference lists of relevant original studies and reviews were manually screened to identify additional literature.

**Fig. 1:**
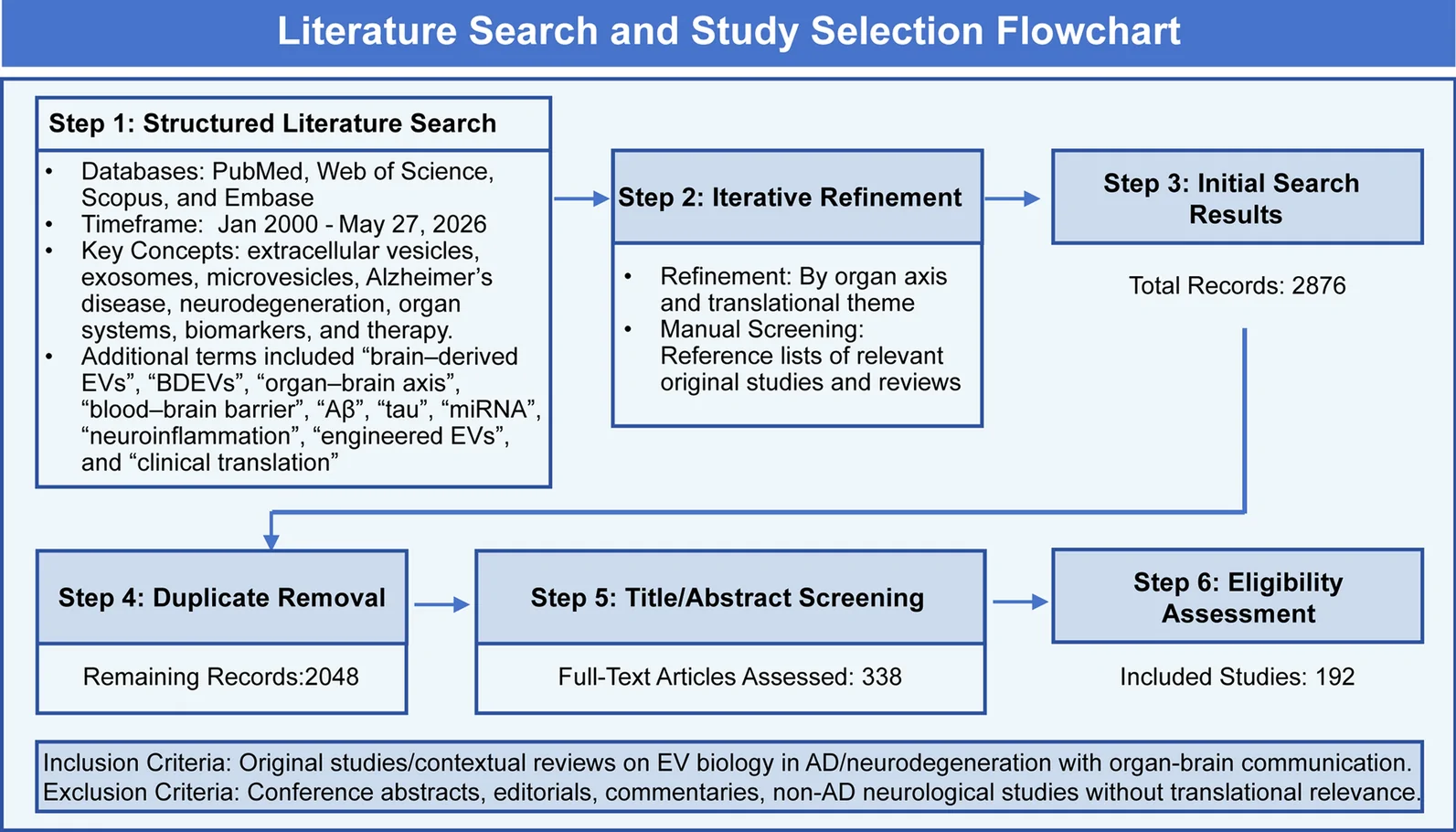
Literature search and study selection flowchart. A structured literature search was conducted using PubMed, Web of Science, Scopus, and Embase, together with manual reference-list screening. The search covered publications from January 2000 to May 27, 2026. After duplicate removal, 2,048 records were screened by title and abstract, 338 full-text articles were assessed for eligibility, and 192 studies were retained as core evidence for this narrative review. EV, extracellular vesicle; AD, Alzheimer’s disease

The initial search identified 2,876 records. After removing duplicates, 2,048 records were screened by title and abstract, of which 338 full-text articles were assessed for eligibility. A total of 192 studies met the inclusion criteria and were retained as core evidence for this narrative review. A detailed flow diagram of the study selection process is provided in Fig. 1.

We prioritized original studies when evaluating mechanistic claims and used review articles primarily for contextual framing. Studies were considered relevant if they addressed EV biology in AD or closely related neurodegenerative contexts with clear relevance to communication between the brain and at least one peripheral organ. Conference abstracts, editorials, commentaries, and studies focused exclusively on non-AD neurological conditions without clear translational relevance were excluded.

## Biological characteristics of EVs

The biological characteristics of EVs underpin both their functional diversity and the interpretive challenges that surround them. This section summarizes the key features of EV biogenesis, classification, cargo composition, and communication mechanisms, while emphasizing a central issue that shapes both experimental interpretation and clinical translation: EV heterogeneity. In the context of AD-related inter-organ communication, this heterogeneity is not merely descriptive; it directly affects source attribution, biodistribution analysis, and causal inference.

### Biogenesis, classification, and core definitions

Extracellular vesicles comprise a heterogeneous population of membrane-enclosed particles released by cells and are commonly classified into three broad subtypes according to biogenesis: exosomes, microvesicles, and apoptotic bodies [25]. Exosomes are typically described as small vesicles of endosomal origin that arise when intraluminal vesicles form within multivesicular bodies and are subsequently released upon fusion with the plasma membrane [26, 27]. In contrast, microvesicles are generated by outward budding of the plasma membrane, whereas apoptotic bodies arise during membrane blebbing and fragmentation in programmed cell death [28]. Although these distinctions are conceptually useful, they are difficult to preserve in most biological isolates, where mixed EV populations are the norm.

**Fig. 2:**
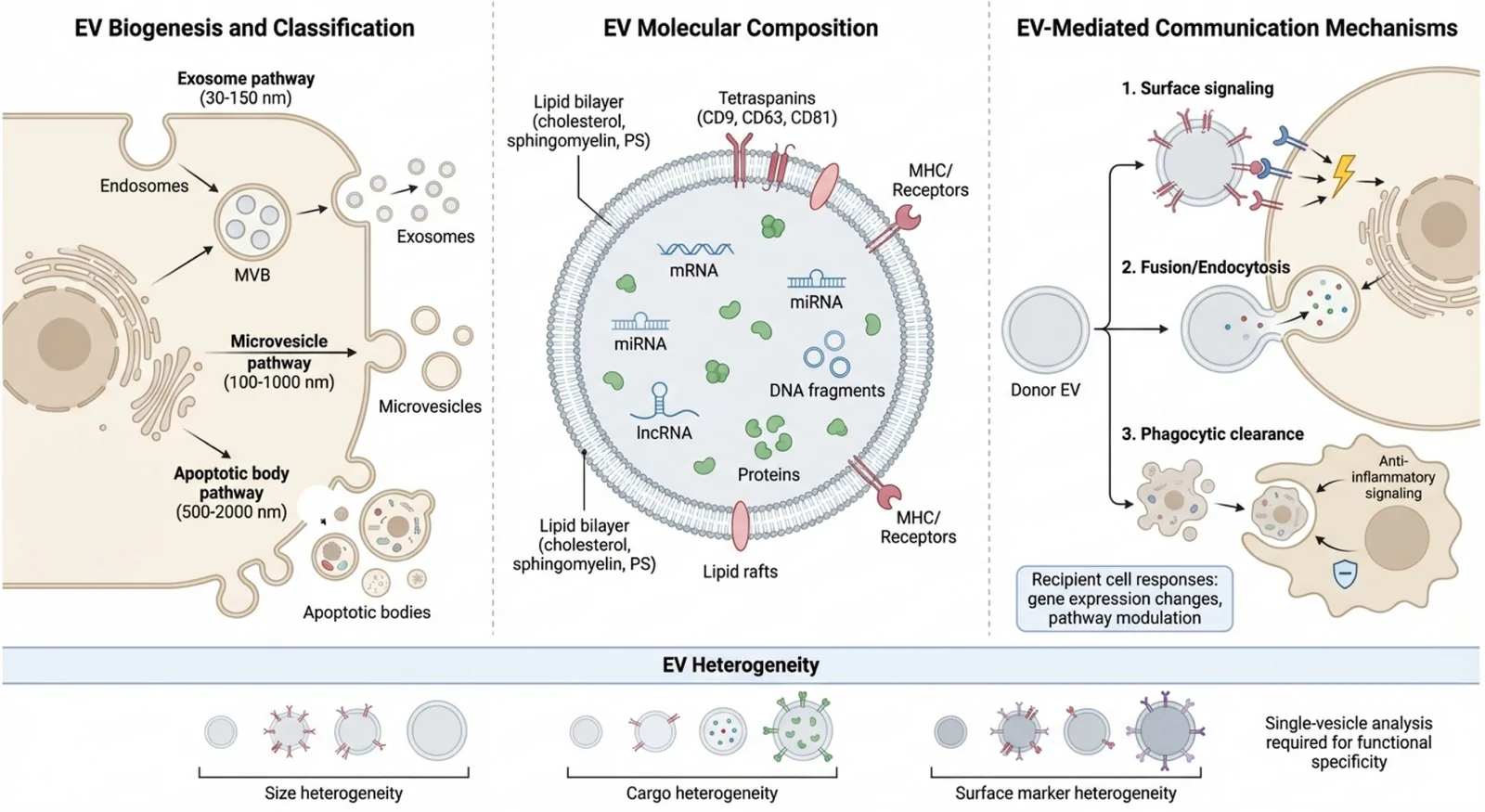
Biological characteristics of extracellular vesicles: from biogenesis to functional heterogeneity. Schematic representation of extracellular vesicle (EV) biogenesis, molecular composition, and mechanisms of intercellular communication. EVs are classified into exosomes, microvesicles, and apoptotic bodies based on their size and biogenesis pathway. Their cargo includes nucleic acids, proteins, and lipids, which are selectively sorted and reflect the physiological state of the donor cell. EVs mediate functional effects in recipient cells through surface signaling or internalization. The inherent heterogeneity of EV populations, arising from diverse cellular origins and biogenesis routes, necessitates single-vesicle analysis to decode functional specificity

Because current isolation approaches rarely recover pure vesicle populations defined by biogenesis alone, origin-based terminology should be applied with caution [14]. Contemporary consensus therefore recommends describing EV preparations using combinations of physical properties (such as size and density), biochemical features (including protein and lipid markers), and, where justified, cellular or tissue enrichment [29]. This issue is especially important in studies of AD-related organ–brain communication, where claims often depend on assigning EVs to a specific organ or cell type from complex biofluids. In this setting, imperfect source attribution can substantially weaken mechanistic conclusions [30–32] (Fig. 2).

### Molecular cargo and physicochemical properties

The functional breadth of EVs derives not only from their cargo composition but also from their physicochemical properties, which together shape stability, circulation time, biodistribution, and interaction with recipient cells [33] (Fig. 2). EV membranes are often enriched in cholesterol, sphingomyelin, and phosphatidylserine, features that can enhance membrane stability and influence recognition at target-cell interfaces [34]. In the context of organ–brain communication, these properties may help determine whether EVs remain intact long enough to reach neurovascular interfaces and whether they preferentially engage endothelial, immune, or neural target cells.

EVs contain a broad range of molecular components. Commonly used protein markers include tetraspanins such as CD9, CD63, and CD81, as well as endosome-associated proteins including TSG101 and ALIX [35]. Beyond these identifiers, EVs may carry functional cargos such as proteins, lipids, metabolites, messenger RNAs, microRNAs, long non-coding RNAs, circular RNAs, and, in some contexts, DNA fragments [36, 37]. Importantly, marker identity should not be conflated with source specificity, and the presence of a molecule within EV isolates does not in itself establish functional relevance [38].

Cargo incorporation is increasingly understood as a selective and regulated process rather than a passive reflection of cytoplasmic content [39, 40]. This selective loading is central to the idea that EVs can function as information carriers across tissues. At the same time, selective enrichment should not be inferred from presence alone: comparative enrichment analyses and functional validation are required before assigning a causal role to a specific EV-associated cargo.

### Communication mechanisms, functional implications, and the challenge of heterogeneity

EVs mediate intercellular communication through several non-mutually exclusive mechanisms, and the biological outcome depends on EV subtype, cargo composition, and recipient-cell context [41] (Fig. 2). One mechanism is surface-mediated signaling, in which EV membrane proteins or lipids engage receptors on target cells without requiring vesicle internalization. A second mechanism involves cargo delivery following EV uptake through membrane fusion or endocytic pathways, enabling transferred proteins, RNAs, or other molecules to modify recipient-cell behavior [42]. A third mechanism is clearance-associated immune modulation, which is particularly relevant for apoptotic bodies and other vesicles recognized by phagocytes. Distinguishing among these mechanisms is important because each implies different experimental requirements for demonstrating causality.

Through these mechanisms, EVs participate broadly in physiological regulation and disease processes [43, 44]. However, a persistent obstacle to interpreting EV function and a key barrier to translation, is their extensive heterogeneity [45]. This heterogeneity spans multiple dimensions. Moreover, even within a single isolate, EVs form a continuum of sizes, densities, and molecular features, likely comprising functionally specialized subpopulations [46, 47]. EVs released by different cell types–or by the same cell type under different states, can differ markedly in abundance, size, surface composition, and cargo. Even within a single preparation, biologically meaningful subpopulations may be diluted by bulk isolation approaches. In plasma and other complex biofluids, co-isolated lipoproteins, protein aggregates, and non-vesicular particles can further complicate cargo interpretation. As a result, causal links between a specific EV feature and an observed phenotype are often less secure than they initially appear.

For these reasons, single-vesicle-resolution approaches and refined fractionation methods are gaining importance [48–51]. These technologies may help resolve mixed organ-origin populations, identify rare but functionally important EV subfractions, and link donor identity more convincingly to recipient targeting and biological effect. In the AD field, such advances will be particularly valuable for testing whether organ-derived EVs truly reach relevant brain interfaces under endogenous conditions and whether specific EV subpopulations are mechanistically distinct.

### Challenges in assigning organ-of-origin to EVs: methodological limitations and confounding factors

Assigning EVs to a specific organ or cell type of origin is a central requirement for testing inter-organ communication hypotheses in AD. However, this remains one of the most challenging methodological problems in the EV field. In complex biofluids such as blood or cerebrospinal fluid, EVs originate from multiple tissues, and no single isolation or detection method currently provides absolute source specificity. The following paragraphs summarise the most commonly used approaches for inferring organ-of-origin, along with their intrinsic limitations and major confounders.

**Donor-cell culture origin.** EVs collected from conditioned media of defined primary cells or cell lines are often used to model organ-specific signals. This approach offers high experimental control and is valuable for proof-of-concept mechanistic studies [52]. However, results obtained in vitro may not reflect the complexity of endogenous EV release, cargo loading, or biodistribution in vivo. Culture conditions (e.g., serum starvation, hypoxia, 2D vs. 3D architecture) can alter EV yield and composition, and the absence of physiological tissue context and immune or vascular components limits extrapolation to whole-organism behaviour.

**Tissue-derived EV preparations.** EVs isolated directly from homogenised tissues (e.g., liver, gut, adipose) retain a more native molecular profile. Nevertheless, tissue-derived isolates contain EVs from all resident cell types (parenchymal, stromal, endothelial, immune), making it impossible to attribute a functional effect to a single cell population without additional fractionation [53]. Furthermore, tissue processing inevitably introduces some degree of cell damage and may co-isolate non-EV particles or intracellular vesicles released during homogenisation [54].

**Immunoaffinity enrichment.** Surface markers (e.g., L1CAM for neuron-derived EVs [55], CD61 for platelet-derived EVs [56]) are widely used to enrich subpopulations from biofluids. While this can increase relative abundance of a putative source, no known marker is absolutely specific to one organ or cell type. For example, L1CAM is also expressed on some peripheral tumours and activated immune cells, and platelet markers may be present on EVs from megakaryocytes [55]. Cross-reactivity, low affinity, and variable epitope accessibility on different EV subpopulations further complicate interpretation. Enrichment should therefore be regarded as probabilistic rather than definitive.

**Genetic / source-tracing approaches.** In animal models, conditional expression of fluorescent or bioluminescent reporters (e.g., CD63-GFP under a tissue-specific promoter) [57] or Cre-LoxP-based trafficking systems can provide high specificity for tracking EVs from a defined cell population [58]. These are currently the gold standard for source-resolved in vivo studies. However, reporter expression may not fully recapitulate endogenous EV biogenesis, and the labelling approach itself can influence vesicle release or cargo sorting. Importantly, results from transgenic models require cautious translation to human physiology.

**Cargo- or marker-based inference.** The presence of a tissue-enriched protein, lipid, or RNA in an EV isolate is often used to infer organ-of-origin [59]. This inference is particularly weak when based on a single analyte, because many molecules are shared across tissues (e.g., apolipoproteins, metabolic enzymes) or can be secondarily adsorbed onto EV surfaces. Even when a cargo is enriched in one organ under steady state, disease-induced changes in expression or barrier permeability can create false positives. Rigorous inference requires comparative enrichment analysis (e.g., higher abundance in the EV fraction than in source tissue homogenate) and functional validation.

Several confounding factors affect all methods of source attribution. Non-vesicular contaminants, including co-isolated lipoproteins (especially in plasma), protein aggregates, and chylomicron remnants, can carry molecular markers identical to those found on genuine EVs and thereby produce artefactual signals [53]. In addition, mixed organ contributions are unavoidable, because a single biofluid sample contains EVs derived from many different organs; without single-vesicle resolution, bulk measurements represent an averaged signal that may be dominated by high-abundance sources rather than the organ of interest. Disease-induced changes further complicate interpretation, as AD and its common comorbidities (e.g., metabolic syndrome, vascular dysfunction) alter EV release profiles, barrier permeability, and cargo composition, which can confound cross-sectional source attribution. Finally, loss of directionality is a critical issue: detection of a brain-enriched marker in plasma does not in itself prove brain-to-periphery trafficking, because the same marker may reflect peripheral expression under pathological conditions or leakage across a disrupted BBB.

In summary, no single method provides definitive organ-of-origin assignment in human studies, and each approach carries distinct limitations and confounding risks. Future progress will depend on combining orthogonal strategies (e.g., immunoaffinity enrichment with single-EV omics and in vivo tracing models) and, most importantly, interpreting source attribution as a probabilistic, hypothesis-generating tool rather than a categorical proof of causal communication (Table 1).

**Table 1:** Summary of methods for inferring EV organ-of-origin limitations and major confounders

Method	Description	Key limitations / confounders
Donor-cell culture origin	EVs collected from defined cell types in vitro	Lacks tissue microenvironment; culture artefacts (serum, hypoxia, matrix); no biodistribution information
Tissue-derived EV preparations	EVs isolated from homogenised organs	Mixed cell-of-origin within tissue; processing damage; co-isolation of intracellular vesicles and debris
Immunoaffinity enrichment	Capture using surface markers (e.g., L1CAM, CD61)	No absolute marker specificity; cross-reactivity; variable epitope exposure; probabilistic enrichment only
Genetic / source-tracing	Transgenic reporters (e.g., CD63-GFP under tissue-specific promoter)	Gold standard in animals but not translatable to humans; reporter expression may alter EV biology
Cargo-/marker-based inference	Single protein, lipid or RNA (e.g., apoE, miR-122)	Shared across tissues; secondary adsorption; disease-induced expression changes; no causality

## The neurovascular interface and perivascular clearance as common gatekeepers of organ-brain EV communication

The BBB has historically been conceptualized as a largely passive, static wall that either permits or denies access of circulating substances to the central nervous system. However, contemporary neurovascular biology demands a more nuanced view: the BBB is not a passive barrier but rather a highly dynamic, multicellular functional module known as the neurovascular unit (NVU) [60]. The NVU comprises cerebral microvascular endothelial cells joined by tight junctions, pericytes embedded within the basement membrane, astrocytic endfeet ensheathing the capillary surface, and adjacent neurons and microglia, all of which collectively determine whether, how, and to what extent peripheral EVs engage the brain [61, 62]. Within this integrated framework, the NVU actively regulates EV contact, sorting, transcellular routing, and clearance rather than simply acting as a passive filter. Consequently, the question of whether peripheral EVs “reach the brain” must be reframed: under what conditions, via which specific NVU-dependent pathways, and with what downstream consequences do peripherally derived EVs gain access to the CNS parenchyma or perivascular compartments [63]?

### Routes of peripheral EV entry into the brain

Peripheral EVs can gain access to the CNS via three anatomically and mechanistically distinct routes, each with different implications for cargo delivery, cell-type engagement, and pathological signaling.

**Receptor-mediated and adsorptive transcytosis.** The most regulated and selective route for EV entry across an intact BBB is transcytosis through cerebral endothelial cells. This process can be either receptor-mediated, wherein specific EV surface ligands engage endothelial receptors such as low-density lipoprotein receptor-related protein 1 (LRP1) [64], the receptor for advanced glycation end-products (RAGE) [65], or the transferrin receptor, triggering vesicular internalization and trafficking to the abluminal membrane; or adsorptive, wherein electrostatic or lectin-based interactions between EV surface glycans or lipids and the endothelial glycocalyx facilitate non-specific uptake and transcellular transport [66]. It is important to emphasize that these receptors represent plausible or candidate endothelial engagement routes, not pathways that have been uniformly validated across all organ-derived EV populations. Nevertheless, the presence of such receptor systems provides a mechanistic basis for selective EV subpopulation trafficking and highlights potential targets for therapeutic engineering (see Sect. “Potential of EVs in disease treatment”).

**Paracellular leakage during barrier injury.** When the BBB is compromised by systemic inflammation, hypertension, metabolic stress, or AD-related vascular pathology, tight junction proteins such as claudin-5, occludin, and ZO-1 become downregulated or mislocalized, creating paracellular gaps [65, 67]. Under these conditions, circulating EVs-regardless of surface receptor profile-may passively enter the perivascular space via paracellular leakage. This route is likely to be quantitatively significant in advanced AD, where BBB breakdown is well documented, but is less likely to dominate in early disease stages [68, 69]. Importantly, paracellular entry bypasses the sorting and selection mechanisms inherent to transcytosis, meaning that the EV populations reaching the brain under conditions of barrier injury may differ substantially from those that cross an intact NVU.

**Choroid plexus and CSF-associated entry.** A third, often overlooked route involves the choroid plexus, a highly vascularized structure located within the cerebral ventricles that lacks a classical BBB and instead forms the blood–CSF barrier [70]. Circulating EVs may enter the CSF compartment via the choroid plexus epithelium, after which they can distribute along CSF flow pathways, enter the brain parenchyma via perivascular spaces, or be cleared via meningeal lymphatic vessels. This route may be particularly relevant for EVs that are too large for efficient transcytosis or those that lack the requisite surface ligands for endothelial receptor engagement.

Critically, regardless of which entry route predominates, peripheral EVs first encounter the cerebral microvascular endothelium and its associated perivascular compartments, not neurons or other deep parenchymal cells. This anatomical reality has profound functional implications: the initial biological responses to peripheral EV signals are mediated by endothelial activation, pericyte signaling, and astroglial endfoot responses, and only secondarily propagate to neuronal and glial populations within the neuropil [71]. Therefore, the cerebral microvascular endothelium serves as the shared convergence point for diverse peripheral EV populations, irrespective of their organ of origin.

### Perivascular and glymphatic clearance: the fate of EVs that enter the brain

**Fig. 3:**
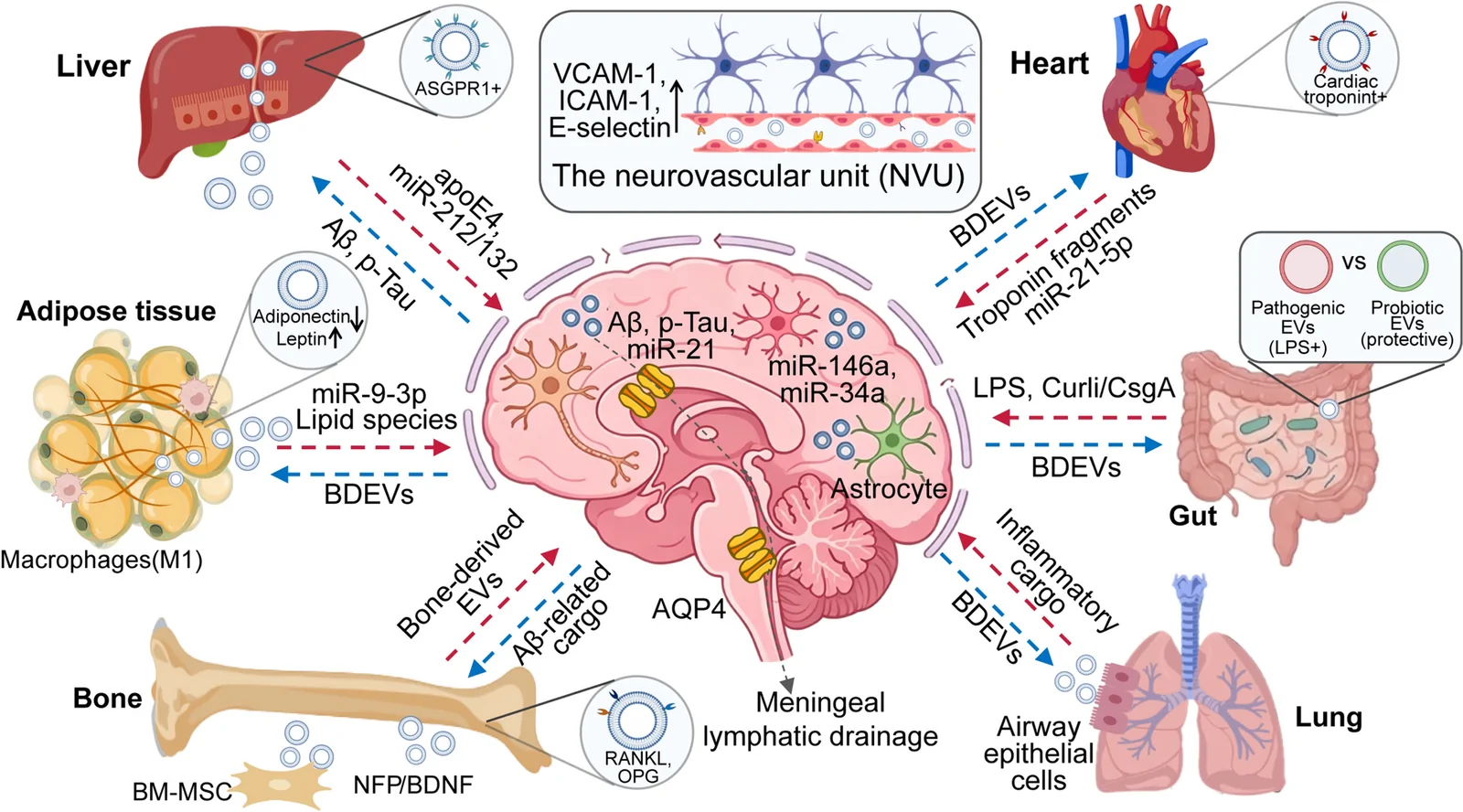
Systemic bidirectional communication via extracellular vesicles in AD: a vascular-centric integrative framework. Peripheral organs—including liver (releasing EVs carrying apoE4, miR-212/132), heart (cardiac troponin fragments, miR-21-5p from M1 macrophages), gut (pathogenic EVs with LPS, Curli/CsgA; probiotic EVs with protective cargo), adipose tissue (EVs enriched in miR-9-3p, leptin, adiponectin, and lipid species), bone (EVs containing RANKL, OPG, BM-MSC-derived NEP/BDNF, and Aβ oligomers), and lung (airway epithelial EVs with inflammatory cargo)—release distinct EV populations that can cross the blood‑brain barrier and converge on the neurovascular unit (NVU), where they trigger endothelial activation (ICAM-1, VCAM-1, E-selectin), pericyte dysfunction, and NLRP3 inflammasome signaling. Simultaneously, brain-derived EVs (BDEVs) carrying astrocytic miR-34a, miR-146a, and other disease-related molecules may exit the CNS and modulate peripheral organs. The NVU serves as the central gatekeeper, with AQP4-dependent glymphatic and meningeal lymphatic pathways governing perivascular clearance. This integrated framework highlights the shared molecular pathways through which peripheral and central EVs influence AD pathology and potential therapeutic targeting

Entry into the CNS is only half the story. Equally important is the question of how-and how efficiently-EVs and their associated cargos are cleared from the brain once they have crossed the NVU. The glymphatic system, an aquaporin-4 (AQP4)-dependent perivascular clearance pathway driven by astrocytic endfoot polarization and CSF-interstitial fluid exchange [72], plays a central role in determining the residence time and signaling consequences of any EV-associated cargo that enters the perivascular space. In the healthy brain, CSF enters along periarterial spaces, mixes with ISF and solutes in a process mediated by AQP4, and exits along perivenous spaces, eventually draining into meningeal lymphatic vessels and cervical lymph nodes (Fig. 3).

Glymphatic dysfunction is increasingly documented in aging and AD, where AQP4 depolarization [73], perivascular drainage failure, and reduced ISF flux impair the clearance of both soluble Aβ and any EV-associated cargo that enters the perivascular compartment. Emerging evidence also suggests that the glymphatic system may serve not only as a clearance pathway but also as a conduit for the long-range transport and signaling of EVs throughout the CNS, further complicating the distinction between clearance and propagation [69, 74]. This dual role implies that glymphatic impairment could both prolong the exposure of neural and glial cells to EV-delivered pathogenic signals and alter the spatial distribution of EV-mediated communication within the brain.

This perspective refines a central mechanistic point: BBB compromise is not only about “letting things in,” but also about “failing to clear them out.” The same peripheral EV that may be harmless under conditions of intact clearance could become disease-relevant when glymphatic outflow is impaired, amplifying neuroinflammation and protein aggregation. Therefore, studies of EV-mediated organ-brain communication should consider not only entry mechanisms, but also the perivascular clearance status of the recipient brain.

### A vascular-centric integrative framework

Building on the above, a vascular-centric integrative model is proposed in which the cerebral microvascular endothelium and the NVU serve as the shared convergence point for diverse peripheral EV signals (Fig. 3). Regardless of whether EVs originate from the liver (apoE4-enriched vesicles), heart (injury-associated EVs), gut (bacterial or host intestinal EVs), lung (environmental exposure-remodeled EVs), bone (osteocyte- or BM-MSC-derived EVs), or adipose tissue (metabolic stress-associated EVs), they must all first engage the same neurovascular interface. This convergence suggests that diverse organ-derived EV populations may activate common downstream mechanisms, including: (i) endothelial activation with upregulation of adhesion molecules (ICAM-1, VCAM-1, E-selectin) [75] and pro-inflammatory cytokine release; (ii) disruption of tight junction proteins (claudin-5, occludin, ZO-1), leading to increased BBB permeability [67]; (iii) activation of canonical inflammatory cascades such as NF-κB and NLRP3 inflammasome signaling [76, 77]; and (iv) secondary pericyte dysfunction and glial (microglial, astrocytic) responses.

Importantly, this convergence framework does not imply that all organ axes are equally established or equally potent; the comparative evidentiary maturity of each axis-as detailed in Sects. "Liver–Brain interplays in AD"–"Adipose–Brain interplays in AD" and summarized in Table 2 remains distinct. Rather, it provides mechanistic coherence by identifying the shared pathways through which multiple peripheral EV signals could influence brain vulnerability. This integrative perspective also directly motivates the therapeutic strategies discussed in Sect. “Potential of EVs in disease treatment”, particularly those aimed at stabilizing the endothelium, blocking shared inflammatory cascades, or restoring perivascular clearance.

**Table 2:** Specific mechanisms of EV-mediated organ–brain communication in Alzheimer’s disease

Organ–Brain axis	EV source	Key cargo	Target cells	Signaling pathway / Functional impact	Level of evidence	Ref
Liver–Brain	Hepatocytes	apoE4	Cerebral endothelial cells (e.g., LRP1)	Inhibits LRP1-mediated Aβ clearance; impairs Aβ efflux across BBB	Direct	[85]
	Hepatocytes	apoE4	Neurons	Proteolytic cleavage generates toxic fragments; impairs mitochondrial function, exacerbates tau pathology	Direct	[145, 146]
Heart–Brain	Stressed cardiomyocytes	Cardiac troponin I/T fragments, ANP precursor fragments	Brain endothelial cells	Activates NF-κB and MAPK pathways; induces endothelial inflammation, disrupts BBB	Inferred	[101, 102]
	Cardiac cells	miR-21-5p	Microglia	Activates pro-inflammatory pathways; induces chronic neuroinflammatory environment	Direct	[100]
Gut–Brain	AD-state microbiota	Various PAMPs (e.g., LPS)	Microglia	Activates NLRP3 inflammasome, triggers pyroptosis; establishes persistent pro-inflammatory state	Direct	[106, 111]
	Pathogens (e.g., *H. pylori*)	Pathogen-associated components	Glial cells, complement system	Compromises BBB via glial activation and complement signaling; triggers neuroinflammation	Direct	[113, 116]
	Gut microbiota	Bacterial amyloid protein CsgA	Brain neurons/interstitium	Acts as cross-seeding agent for Aβ aggregation; promotes aberrant amyloid deposition	Direct	[120]
	*S. paucimobilis*	Regulatory cargo	Neurons/glia	Upregulates MeCP2; modulates neurotrophic support and inflammation; counteracts Aβ toxicity	Direct	[121]
Lung–Brain	Airway epithelial cells	Inflammatory signal cargo	Hippocampal neurons/glia	Carries inflammatory signals across biological barriers; triggers/exacerbates neuroinflammation	Inferred	[124]
Bone–Brain	AD BDEVs	Aβ oligomers	Osteoclast precursors	Aβ acts directly on bone microenvironment; enhances osteoclast differentiation and activity	Inferred	[131]
	BM-MSCs	Neprilysin (NEP), BDNF	Neurons, glia	Degrades Aβ; provides neurotrophic support; attenuates neuroinflammation; reduces Aβ plaque burden	Direct	[132]
	Osteocytes	Factors for energy metabolism & Aβ degradation	Neurons, vasculature	Improves mitochondrial function; combats oxidative stress; improves cognitive function	Direct	[134]
Adipose–Brain	Adipocytes (obese individuals)	Specific lipid profile (LPC, SM subtypes)	Aβ peptides	Modulates Aβ aggregation kinetics; aberrantly promotes Aβ fibril formation	Direct	[19]
	Pathological adipocytes	miR-9-3p, miR-6760-3p	Neurons	Downregulates BDNF; inhibits CREB signaling; impairs synaptic plasticity and neurogenesis	Direct	[141, 142]
	AD-MSCs	Neprilysin (NEP)	Cerebral Aβ plaques	Directly degrades Aβ; facilitates clearance of Aβ plaques	Direct	[144]

Level of EV-specific evidence classification.

Direct: Evidence derived from studies explicitly demonstrating that the molecular cargo is carried by EVs and that EV-mediated transfer is responsible for the described functional effect.

Inferred: Evidence based on studies showing association of the molecular cargo with EVs or EV-enriched fractions, but where definitive proof of EV-specific causality (e.g., EV depletion, EV-specific inhibitor, or purified EV transfer experiments) is incomplete or partially confounded by non-vesicular components.

Collectively, the NVU and the glymphatic/lymphatic clearance apparatus constitute the central gatekeepers that determine the net biological impact of peripheral EVs on the brain. Clarifying how specific EV subpopulations engage, cross, or are cleared by these systems represents a critical frontier for understanding organ–brain communication in AD and for developing targeted interventions that interrupt maladaptive signaling while preserving physiological exchange.

## Bidirectional communication of EVs in the brain and other organs

Inter-organ communication depends on the transfer of biological information across tissues and cell types. EVs contribute to this exchange by transporting proteins, lipids, and nucleic acids through biofluids and by engaging recipient cells via surface interactions and cargo delivery. Within the CNS, neurons, microglia, and astrocytes release EVs in response to physiological cues and, more prominently, under stress or pathological conditions [21, 78]. A subset of BDEVs has been reported to enter systemic circulation and interact with peripheral tissues, suggesting a plausible route for CNS-to-periphery signaling. However, the endogenous magnitude, destination specificity, and physiological significance of this trafficking remain insufficiently quantified.

Communication is also bidirectional: peripheral organs may influence the brain through EVs released during metabolic stress, inflammation, or organ dysfunction. Such EVs may gain greater access to neurovascular interfaces when blood–brain barrier (BBB) integrity is compromised, thereby contributing to neuroinflammation, vascular dysfunction, or altered Aβ/tau-related processes [79]. Notably, the current evidentiary balance in the literature strongly favours periphery-to-brain EV signaling, whereas evidence for functionally significant brain-to-periphery EV pathways remains substantially weaker, more indirect, and largely restricted to a few experimental contexts. The following subsections therefore evaluate individual organ axes with explicit attention to this asymmetry, distinguishing well-supported peripheral-to-central mechanisms from speculative or insufficiently quantified reverse directions [80, 81].

### Liver–brain interplays in AD

The strongest support for a liver–brain contribution to AD lies in the well-established links between hepatic metabolic or inflammatory dysfunction and AD risk, together with the liver’s central role in systemic lipid handling and peripheral Aβ clearance. Within this framework, EVs are best considered one candidate signaling layer through which liver state may be translated into brain-relevant consequences. When metabolic stress, systemic inflammation, or increased circulating abnormal protein burden occurs, hepatocytes and non-parenchymal liver cells may remodel EV release and cargo composition [82]. However, EV-mediated effects should be interpreted alongside, rather than as replacements for, established hepatic metabolic and inflammatory pathways.

Liver-derived EVs have been proposed as carriers of apoE-related signals with potential relevance to Aβ metabolism. Under pathological conditions, hepatocytes have been reported to release increased EVs enriched in apoE isoforms, including apoE4 [83]. These vesicles may contain apoE-associated lipid components and display liver-associated surface features [84]. At present, the most defensible interpretation is that liver-derived EVs could influence AD-relevant biology through several testable routes: by modulating receptor-mediated Aβ transport at the neurovascular interface, by altering systemic lipid environments that secondarily affect vascular and amyloid biology, or, under conditions of impaired barrier integrity, by delivering apoE-related signals to CNS-adjacent compartments [85]. Importantly, these mechanisms remain inferential unless studies quantify endogenous delivery, identify recipient cell types, and distinguish EV-associated apoE effects from those of abundant circulating lipoproteins and free apoE.

Because apoE4 is already known to alter Aβ binding, receptor-mediated trafficking, and neuronal vulnerability, it is biologically plausible that apoE4-associated liver EVs could contribute to AD-relevant processes [86, 87]. However, it is important to note that apoE4-associated BBB dysfunction has also been demonstrated independently of EVs-through pericyte degeneration, tight junction loss, and other vascular abnormalities. Acknowledging this helps the reader understand what EVs actually add to the picture versus what is already explained by lipoprotein-mediated or cell-autonomous mechanisms. Therefore, liver-derived EVs should be interpreted as an additional candidate signaling layer, not as a replacement for established apoE/lipoprotein‑mediated biology. These downstream consequences should not be assumed to reflect liver-EV delivery unless directly demonstrated in vivo. Likewise, EVs carrying apoE2 or apoE3 may be neutral or potentially protective with respect to Aβ handling, but their net impact and delivery efficiency remain unresolved [88–90]. Thus, apoE-related liver EV signaling is best framed as a plausible but still under-resolved mechanism that may complement, rather than substitute for, well-characterized EV-independent vascular and metabolic pathways. Beyond apoE-related biology, inflammatory remodeling of liver-derived EVs may provide a broader route through which hepatic state influences brain vulnerability. Chronic inflammatory inputs from the gut or systemic circulation may alter Kupffer cell activation and thereby reshape EV release and cargo composition [91–94] (Fig. 3).

Overall, liver-derived EVs are best positioned as candidate conduits linking peripheral metabolic and inflammatory states to AD-relevant neurovascular and neuroimmune processes, while quantitative trafficking, recipient-cell identification, and EV-specific causality remain key unresolved issues. Regarding the reverse direction-from brain to liver-the current evidence remains substantially less developed. While it has been proposed that BDEVs may contribute to multi-organ communication in neurodegenerative diseases, the specific mechanisms by which BDEVs mediate crosstalk between the central nervous system and peripheral organs, including the liver, remain underexplored [95]. Indeed, research on how EVs facilitate bidirectional communication between the liver and the brain is still considered to be in its nascent stages [94, 96]. AD BDEVs enriched in Aβ- or tau-related cargos have been hypothesized to affect hepatic metabolic or detoxification functions, but this remains a hypothesis-supported rather than a demonstrated causal pathway. No quantitative trafficking studies have established that endogenous BDEVs reach the liver at functionally relevant levels in AD, nor have recipient-cell uptake or EV-specific perturbation experiments been performed to validate this direction. Therefore, while the possibility of brain-to-liver EV signaling is conceptually interesting, it should be interpreted as a speculative, incompletely resolved hypothesis rather than an established component of inter-organ communication in AD (Table 2).

### Heart–brain interplays in AD

Within the broader comorbidity framework linking AD and cardiovascular disease, the heart has been proposed to communicate with the brain through EV-mediated signaling [97]. Cardiovascular risk factors and heart failure can remodel EV secretion and cargo composition in cardiomyocytes, fibroblasts, and resident immune cells [98, 99]. However, because many cardiovascular contributions to cognitive decline can already be explained by reduced perfusion, hypoxia, and systemic inflammation, the key question is not whether the heart influences the brain, but whether EVs provide additional mechanistic specificity and therapeutic tractability beyond these established pathways [100] (Fig. 3, Table 2).

Cardiac EVs have been proposed to access neurovascular interfaces through the circulation, particularly under conditions of BBB vulnerability [101]. Mechanistically, the most plausible interpretation is that cardiac injury-associated EV cargo may engage the cerebral microvascular endothelium and perivascular compartments, leading to a coordinated neurovascular response. Rather than a simplified endothelial-to-glial sequence, the downstream cascade should be understood as involving capillary-level neurovascular changes: (i) endothelial activation with upregulation of adhesion molecules (e.g., ICAM-1, VCAM-1) and release of pro-inflammatory mediators; (ii) pericyte involvement and dysregulation, including detachment or dysfunction that compromises capillary integrity and flow regulation; and (iii) secondary astroglial and microglial responses that amplify inflammation and may contribute to synaptic dysfunction. In this framework, the neurovascular unit acts as the initial site of exposure, but the pathological cascade spreads through the entire NVU, not solely along an endothelial-to-glial axis [102].

In myocardial infarction models, heart-derived EVs enriched in miR-21-5p have been reported to reach the brain and promote pro-inflammatory microglial responses [100]. These findings are informative but should be bounded to the specific injury context, particularly because acute systemic inflammation may contribute substantially to the observed phenotype. For the reverse direction about brain-to-heart EV signaling evidence is even more limited. Whether AD BDEVs meaningfully alter cardiomyocyte metabolism, electrophysiology, or cardiac inflammation remains unresolved and should be framed as a testable question rather than an established reverse pathway. Currently, no direct in vivo studies have quantified endogenous brain-derived EV uptake by cardiac cells or demonstrated functional cardiac consequences in AD models [103, 104].

Overall, cardiac EVs may serve as biomarkers of cardiovascular stress and as plausible contributors to neurovascular and neuroimmune changes in AD-related contexts. However, current evidence does not yet justify strong claims that cardiac EVs directly drive AD progression under endogenous conditions. Quantitative trafficking studies, recipient-cell target engagement, and EV-specific perturbation experiments will be needed to establish causal relevance.

### Gut–brain interplays in AD

Among the organ axes discussed in this Review, the gut–brain axis currently has some of the strongest convergent support for EV-related involvement in AD-relevant biology. In particular, bacterial extracellular vesicles (bEVs) and host intestinal EVs have emerged as plausible mediators through which microbial dysbiosis, intestinal barrier dysfunction, and local inflammation may influence the brain [105]. These vesicles can carry bioactive cargos such as lipopolysaccharide, amyloid-like proteins, small RNAs, and metabolites, making them mechanistically attractive candidates for linking peripheral immune or metabolic disturbances to neuroinflammation and proteinopathy. However, their endogenous exposure levels, disease-stage specificity, and quantitative contribution in human AD remain incompletely defined [106] (Fig. 3, Table 2).

Gut-derived EVs can be broadly divided into host-derived vesicles released from intestinal epithelial, enteroendocrine, or immune cells, and microbiota-derived vesicles released by commensal or pathogenic microbes [107]. Under dysbiosis or impaired gut-barrier conditions, the composition and abundance of both EV classes may change, potentially altering neuroimmune signaling through circulatory or neural routes [108, 109]. Microglia are often proposed as major target cells in this context, particularly in studies suggesting inflammasome activation, pyroptotic signaling, or reinforcement of Aβ-associated inflammation [110].

Little attention has been paid to the reverse direction of brain-to-gut EV signaling. While it is theoretically possible that AD BDEVs could influence intestinal barrier function, gut microbiota composition, or enteric nervous system activity, current evidence is essentially absent. Therefore, the gut–brain axis in AD should be understood predominantly as a periphery-to-central pathway, with the brain-to-gut direction remaining an open, largely unexplored question [111].

A substantial portion of the literature focuses on microbial EVs as immunologically active particles capable of carrying pathogen-associated molecular patterns and other inflammatory signals [112–118]. In experimental systems, EVs derived from organisms such as Helicobacter pylori or Porphyromonas gingivalis have been linked to glial activation, complement signaling, neuroinflammation, and cognitive impairment-related phenotypes. These findings support biological plausibility, but they should not be generalized as definitive evidence that microbial EVs are major endogenous drivers of AD in humans. Administration route, dose, microbial composition, and host barrier state all critically influence interpretation [119].

The possibility that bacterial amyloid-like proteins carried by bEVs may cross-seed Aβ aggregation is conceptually compelling and biophysically plausible [120]. Nevertheless, this mechanism is still supported primarily by indirect or model-based evidence, and its in vivo relevance depends on exposure level, timing, and host susceptibility. By contrast, several studies also suggest that probiotic- or commensal-derived EVs may exert neuroprotective effects by modulating metabolites, inflammatory tone, or neurotrophic signaling [121]. Taken together, these findings indicate that the EV channel itself is not inherently harmful or beneficial; rather, its biological effect depends on cargo composition, microbial context, and host barrier and immune status [122, 123].

Overall, gut-derived EVs are best viewed as candidate mediators linking intestinal state to brain vulnerability in AD. Their plausibility is supported by a convergence of microbiome, inflammation, and preclinical EV studies, but causal contribution in humans remains unproven. Future work should prioritize source-resolved in vivo quantification, stage-specific trafficking analysis, and perturbation-based tests that distinguish EV effects from broader microbial or inflammatory confounders (Fig. 3).

### Lung–brain interplays in AD

The lung is a major interface for environmental exposure and inflammatory input, making it biologically plausible that pulmonary states could be encoded into circulating EV signals with downstream relevance to brain vulnerability. In the current literature, lung–brain EV interactions are described in three distinct but interconnected contexts: (i) environmental exposure-driven remodeling of EV biogenesis and cargo, (ii) endogenous pathophysiological signaling, and (iii) engineered EV-based therapeutic delivery [124]. These contexts should be interpreted separately, because successful brain delivery by engineered EVs does not establish the existence or importance of endogenous lung–brain EV trafficking in AD (Fig. 3, Table 2).

Environmental exposures as upstream determinants of pulmonary and systemic EV remodeling. Inhaled particulate matter (e.g., PM2.5), nanoplastics, and viral particles can directly alter EV biogenesis, cargo sorting, and release from airway epithelial cells, alveolar macrophages, and pulmonary endothelium [125]. Substantial evidence indicates that PM2.5 exposure drives endothelial mitochondrial dysfunction, aryl hydrocarbon receptor (AhR)-mediated inflammatory cascades, and downstream BBB disruption [126]. These processes are directly relevant to EV composition: stressed pulmonary cells release EVs enriched in pro-inflammatory lipids, oxidative stress-related proteins, and regulatory RNAs that can enter the circulation and potentially reach neurovascular interfaces [127]. Air pollution is now a well-recognized risk factor for AD in environmental epidemiology [128], yet the EV-mediated signaling layer that could link inhaled exposures to brain pathology remains largely unexplored. In the revised framework, it is explicitly acknowledged that environmental inputs represent an upstream, physiologically relevant determinant of EV-mediated lung–brain crosstalk, rather than treating EV trafficking as the sole entry point [129].

Endogenous pathophysiological EV signaling. Experimental reports have described airway epithelial EVs entering the brain after intranasal administration in viral-mimetic or inflammatory settings [129]. While these findings are useful proof-of-concept observations, they should be framed as route-specific administration studies rather than definitive evidence that endogenous lung-derived EVs naturally reach the hippocampus or other brain regions at disease-relevant levels. Administration route, formulation, and dose all strongly affect biodistribution. Moreover, even if inhaled exposures alter EV cargo, direct evidence that such EVs quantitatively reach the brain and causally contribute to AD pathology remains limited. Therefore, the lung–brain EV axis is best interpreted as a hypothesis-supporting area with strong biological plausibility, rather than a well-established endogenous pathogenic pathway.

Reverse direction and engineered platforms. Evidence for reverse brain-to-lung EV signaling is even scarcer. Although it is conceivable that BDEVs could influence pulmonary immune or inflammatory states, particularly under conditions of systemic inflammation or barrier disruption, no direct studies have addressed this possibility in AD. A separate body of work has used lung-derived or immune-cell-derived EVs, including neutrophil-associated EV systems, as engineered therapeutic delivery platforms [130]. These studies are best discussed as translational engineering strategies rather than evidence for endogenous pulmonary pathogenic EV signaling. Claims such as rapid hippocampal entry, low immunogenicity, or efficient BBB traversal should be presented as context-dependent properties of specific experimental systems rather than general features of lung-derived EVs.

Overall, the lung–brain EV axis is conceptually compelling and gains additional support from environmental exposure biology, but direct endogenous EV-specific evidence in AD remains comparatively limited. Future studies should explicitly separate (i) environmental exposure-driven EV remodeling, (ii) endogenous trafficking questions, and (iii) therapeutic engineering paradigms (Table 2).

### Bone–brain interplays in AD

Bone is increasingly recognized as an endocrine and metabolic organ, and EVs may provide an additional carrier layer for skeletal signals with potential relevance to brain health. In discussing this axis, it is important to distinguish pathological bone-derived EV signaling from MSC- or osteocyte-derived therapeutic EV applications, because these two lines of evidence rest on different experimental foundations.

Bone-derived EVs released by osteoblasts, osteoclasts, osteocytes, or marrow-associated cells may change in abundance and cargo composition with aging, inflammation, inactivity, or altered bone remodeling [20, 131] (Fig. 3). These vesicles have been proposed to reach the brain via the circulation, but direct endogenous trafficking evidence remains limited. At present, the bone–brain EV axis is conceptually attractive but still relatively under-resolved compared with more extensively studied metabolic axes.

Brain-derived EV effects on bone in the reverse direction have received more attention, but the evidence should be interpreted cautiously. Associations between Aβ-related pathology and altered bone remodeling support a potential brain–bone relationship, but they do not by themselves establish EVs as the principal carrier mechanism [20]. Statements implying that BDEVs enter bone and directly alter remodeling should therefore be downshifted unless direct bone-cell uptake and causal perturbation are demonstrated. More generally, protein-associated effects and EV-mediated delivery should not be treated as interchangeable.

On the therapeutic side, several reports suggest that BM-MSC-derived or osteocyte-derived EVs may support cognition by promoting Aβ degradation, enhancing neurotrophic support, improving mitochondrial function, or mitigating oxidative stress [132–134]. These findings can be grouped into three broad effect classes: neurotrophic/Aβ-clearance support, vascular-metabolic support, and oxidative stress modulation. Such framing is preferable to listing many isolated examples, because it better highlights the major biological themes while keeping model-specific effects in context. Importantly, these benefits remain bounded to specific EV sources, preparation methods, and animal or in vitro systems [135–137].

Overall, bone-derived EVs are plausible mediators linking skeletal immune-metabolic states with CNS health, whereas MSC- and osteocyte-derived EVs also represent therapeutic candidates. However, directionality, in vivo trafficking, recipient-cell uptake, and EV-specific effects beyond classical endocrine or inflammatory signaling remain major unresolved issues [138, 139] (Table 2).

### Adipose–brain interplays in AD

Adipose tissue is both an endocrine and immune organ and is tightly linked to systemic metabolic inflammation. In obesity- and insulin resistance-associated states, adipose-derived EVs have been proposed as transferable carriers that encode peripheral metabolic and inflammatory stress for delivery to distant tissues, including the brain [140] (Fig. 3, Table 2). A central interpretive challenge is determining whether these EVs exert effects beyond those attributable to soluble cytokines, lipid mediators, and co-isolated non-vesicular particles.

Adipose-derived EVs released under metabolic stress may contain altered lipid species, inflammatory mediators, and regulatory RNAs. Recent lipidomic observations suggest that EVs from obese individuals can modulate Aβ aggregation kinetics in vitro in a lipid-type and concentration-dependent manner [19]. These findings are intriguing but should be explicitly framed as biophysical observations in experimental systems rather than proof that adipose EV lipids accelerate cerebral amyloid pathology in vivo.

For RNA cargo, the literature can be more clearly organized around a central mechanistic theme: adipose-derived EV miRNAs may impair neuronal survival, synaptic plasticity, and neurogenesis through suppression of BDNF- and CREB-related signaling [141]. Transfer studies in animal models suggest that EVs derived from metabolically compromised adipose tissue can induce cognitive and synaptic phenotypes in recipient animals [142]. In parallel, patient-derived studies report adipocyte EV miRNA signatures associated with CREB pathway perturbation in AD [143]. These human observations, however, are largely cross-sectional and should not be interpreted as evidence of directionality or causation.

The reverse direction, brain-to-adipose EV signaling, has not been systematically investigated. While it is possible that AD BDEVs could influence adipocyte metabolism, inflammation, or insulin sensitivity, no direct experimental evidence currently supports this hypothesis. This direction should therefore be considered a largely unexplored area rather than an established component of the adipose–brain axis.

In contrast to the pathogenic framing above, EVs derived from healthy or conditioned adipose-derived mesenchymal stem cells are often discussed as cell-free therapeutic tools. These EVs have been reported to carry Aβ-degrading enzymes such as neprilysin and to deliver anti-inflammatory or neurotrophic microRNAs capable of modulating microglial activation and supporting repair [144]. This therapeutic potential can be retained in the manuscript, but it should be accompanied by explicit translational caveats regarding batch consistency, brain delivery efficiency, target specificity, and safety.

Overall, adipose-derived EVs are among the more compelling candidate mediators linking peripheral metabolic dysfunction to CNS vulnerability in AD. The evidence supports biological plausibility and model-dependent transfer effects, but strong claims that adipose EVs directly drive AD progression should be avoided unless EV-specific causality is demonstrated under controlled confounding and physiologically relevant exposure conditions.

## Potential of EVs in disease treatment

EVs, owing to their inherent biocompatibility, ability to engage the neurovascular unit and access brain-related compartments via NVU-dependent routes, and central role in intercellular communication, have emerged as a highly promising platform for diagnosis and therapy in AD [147, 148]. Bridging organ–brain communication to translational applications. The organ-axis framework developed in Sect. "Bidirectional communication of EVs in the brain and other organs" motivates translational EV research in three conceptually distinct but complementary ways. First, it suggests that EVs enriched for specific organ-of-origin signatures could serve as minimally invasive biomarkers of peripheral contributions to AD pathology, provided that source attribution can be sufficiently resolved [149]. Second, it raises the possibility of therapeutically interrupting maladaptive endogenous EV communication-for example, by reducing pathogenic EV release from the liver, gut, or adipose tissue, or by blocking their uptake at neurovascular interfaces. Early proof-of-concept studies have demonstrated that targeting critical components of the ESCRT machinery responsible for EV biogenesis, such as microglial Tsg101, can suppress EV-mediated propagation of tau pathology and ameliorate disease phenotypes in tauopathy mouse models [150]. Third, it informs the rational design of engineered EV therapeutics by leveraging shared mechanisms of neurovascular engagement, cargo delivery, and clearance (glymphatic/perivascular) that are common to both endogenous and exogenous EV pathways [147]. The following subsections discuss diagnostic and therapeutic EV strategies with explicit attention to whether they derive from organ-axis biology (source-informed or pathway-targeting) or from engineering platforms whose producer-cell origin is chosen primarily for manufacturability, delivery efficiency, or immunological properties. Distinguishing these two streams is essential to avoid overstating the maturity of endogenous EV-targeting interventions.

### Diagnostic and prognostic biomarkers

Because EVs carry molecular signatures that reflect the physiological and pathological states of their parent cells, EVs recovered from cerebrospinal fluid or peripheral blood have been widely proposed as minimally invasive "liquid biopsy" readouts of CNS pathology in AD. Importantly, the organ-axis framework outlined in Sect. "Bidirectional communication of EVs in the brain and other organs" suggests that the most informative EV biomarkers may not be those that simply measure total circulating EVs, but rather those enriched for specific tissue-or organ-of-origin signatures, such as brain-derived, liver-metabolic, adipose-inflammatory, or gut/microbiota-associated signals-that reflect defined peripheral contributions to AD pathophysiology. In principle, this enables longitudinal monitoring of disease-relevant molecular changes that would otherwise be inaccessible without direct brain sampling [151]. A major attraction of EV-based biomarker strategies is that they may preserve otherwise labile cargos, while also offering partial enrichment for tissue- or cell-associated information. At the same time, it is important to note that enrichment-based approaches should generally be interpreted as probabilistic rather than absolutely cell-type-pure.

**Fig. 4:**
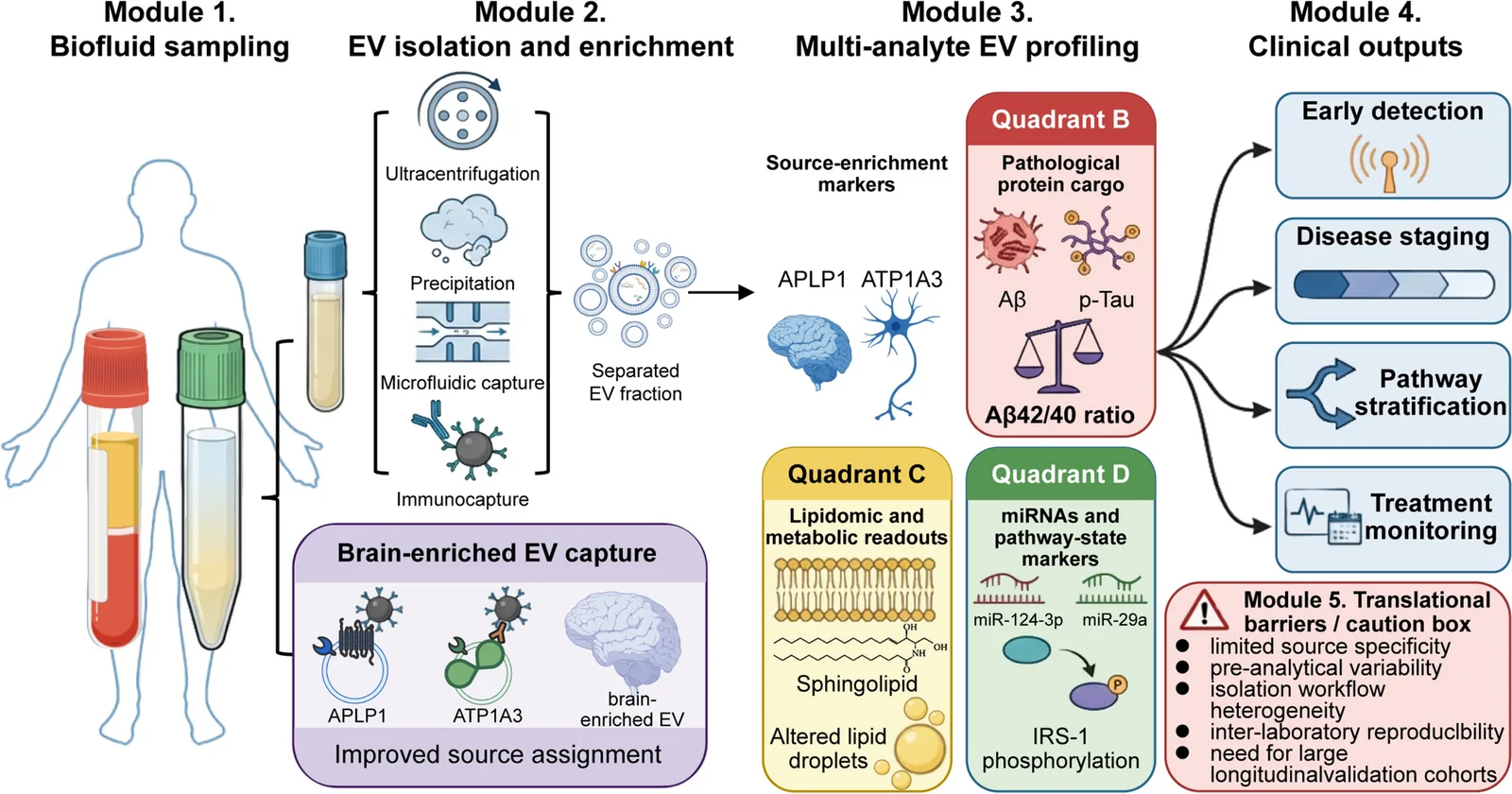
Workflow for EV-based multi-analyte profiling from biofluids for Alzheimer’s disease (AD) clinical applications. The schematic outlines the sequential process from (Module 1) biofluid sampling to (Module 2) EV isolation and enrichment, followed by (Module 3) multi-analyte EV profiling. (Module 4) Clinical outputs include early detection through quadrant-based multi-modal markers: source-enrichment markers (e.g., APLP1, ATP1A3), pathological protein cargo (Aβ, p-Tau, Aβ42/40 ratio), lipidomic/metabolic readouts, and miRNA pathway-state markers (miR-124-3p, miR-29a), as well as treatment monitoring. (Module 5) Key translational barriers and considerations are highlighted, including source specificity, pre-analytical variability, isolation heterogeneity, and the need for standardized protocols and large longitudinal cohorts

Current EV-based biomarker efforts in AD can be organized into four major categories. First, cell-type-enrichment strategies aim to improve the specificity of brain-associated EV capture from peripheral blood. Recent studies suggest that APLP1 and ATP1A3 may offer greater specificity for brain- or neuron-enriched EV fractions than the historically used L1CAM-based approach, thereby improving confidence in cerebral origin assignment [152, 153]. These strategies are directly motivated by the source-attribution challenges discussed in Sect. "Challenges in assigning organ-of-origin to EVs: methodological limitations and confounding factors", and they represent a translational effort to convert organ-axis biology into clinically accessible readouts (Fig. 4). Second, EV-associated pathological proteins-including Aβ42, phosphorylated tau (p-tau), and ratios such as Aβ42/40-have been explored as disease-related markers that may be less vulnerable than free plasma analytes to degradation or dilution effects [154]. These protein measurements are promising, but they should not be assumed to provide a direct one-to-one readout of brain plaque or tangle burden without further validation.

Third, EV-associated metabolic and lipidomic features may capture disease-relevant membrane remodeling and bioenergetic disruption. Notably, BDEVs isolated from AD frontal cortex have been reported to reveal lipid dysregulation with greater sensitivity than bulk tissue lipid profiling, including changes in glycerophospholipids, sphingolipids, plasmalogen phosphatidylethanolamines, polyunsaturated fatty-acyl content, and sphingomyelin/ceramide remodeling [155, 156]. These findings support the idea that EV lipidomics may capture a biologically enriched layer of disease information, although analytical reproducibility and workflow standardization remain major prerequisites for clinical translation. Fourth, regulatory nucleic acids and pathway-state markers have attracted increasing attention. Exosomal miRNAs such as miR-124-3p and miR-29a have been associated with neuroinflammation, synaptic plasticity, and disease progression [157, 158]. In addition, the phosphorylation status of IRS-1 in neuron-enriched EV fractions has been proposed as a non-invasive proxy for brain insulin signaling impairment, a biologically important but clinically difficult domain to assess directly in living patients (Fig. 4).

Despite this promise, EV biomarker translation remains constrained by substantial methodological barriers. These include non-standardized isolation and purification workflows, variable definitions of “brain-derived” or “neuron-derived” EV fractions, limited inter-laboratory reproducibility, the need for highly sensitive yet scalable detection platforms, and insufficient validation in large, diverse, longitudinal cohorts [152, 159] (Fig. 4, Table 3). Pre-analytical variables-including sample handling, storage, freeze–thaw exposure, and capture methodology, may also substantially affect readouts. Accordingly, future EV biomarker pipelines will likely require the combined use of more specific origin-enrichment strategies, multi-analyte biomarker panels, rigorous pre-analytical control, and clinically meaningful benchmarking against existing blood-based AD biomarkers. Under these conditions, EV-based assays may eventually contribute not only to diagnosis, but also to disease staging, pathway stratification, and treatment monitoring.

**Table 3:** Representative EV-based biomarker strategies in Alzheimer’s disease

Key molecule	Specific mechanism / Strategy	Target	Translational maturity	Ref
APLP1, ATP1A3	Enrich BDEVs from peripheral blood via neuron-specific surface markers	Higher specificity than the traditional marker L1CAM, enabling more reliable isolation of brain-derived vesicles	Exploratory	[152, 153]
Glycerophospholipids, sphingolipids	Detecting AD-associated lipid dysregulation	EV lipid profiling is more effective than global brain tissue lipidomics in detecting AD-related lipid disturbances	Exploratory	[155]
Phosphorylation status of IRS-1	Non-invasive assessment of brain pathway status by analyzing signaling molecules in Neuron-Derived EVs	Enables evaluation of insulin resistance and dysfunction in brain insulin signaling pathways in living patients	Exploratory	[151]
miR-124-3p, miR-29a	Detecting regulatory miRNAs carried by EVs as potential diagnostic and prognostic indicators	Closely associated with neuroinflammation, synaptic plasticity, and disease progression in AD	Exploratory	[157, 158]

### EVs as therapeutic carriers and bioactive therapeutic products

**Fig. 5:**
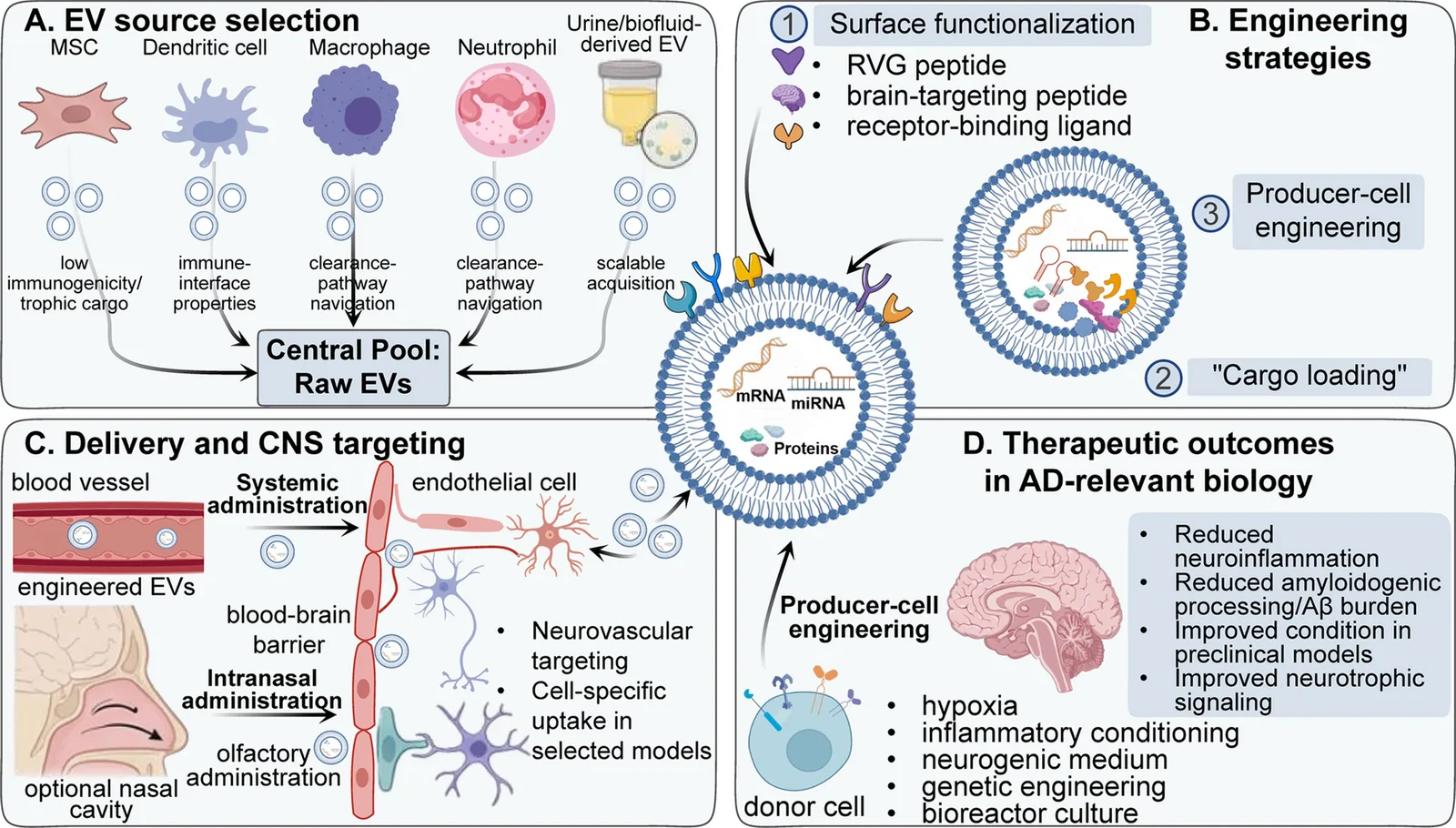
Schematic diagram of engineered EV design and application for AD. The schematic outlines the workflow for engineering EVs, starting from **A** various cellular and biofluid sources. **B** Engineering strategies include surface functionalization with targeting moieties (e.g., RVG peptide) and scalable acquisition methods. **C** Delivery to the CNS is achieved via systemic administration or intranasal/olfactory routes, with engineered EVs accessing brain compartments through NVU-dependent mechanisms (transcytosis, paracellular leakage, or choroid plexus pathways). **D** The therapeutic outcomes in AD models include reduced neuroinflammation, decreased amyloid pathology, and improved neurotrophic signaling

EVs-particularly those derived from mesenchymal stromal/stem cells (MSCs)-are increasingly being investigated both as therapeutic carriers and, in some contexts, as bioactive therapeutic products in their own right. Their appeal lies in their relative biocompatibility, capacity to protect fragile cargos, and potential to engage multiple disease-relevant pathways simultaneously rather than acting through a single molecular target. However, these advantages should be framed as context-dependent rather than universal, because EV source, purification method, loading strategy, and route of administration all substantially influence therapeutic performance (Fig. 5). It is important to distinguish two conceptually distinct therapeutic paradigms that are often conflated in the literature. The first paradigm-inspired by organ–brain biology-aims to interrupt or redirect maladaptive endogenous EV communication (e.g., by blocking pathogenic EV release from the liver or gut, or by neutralizing disease-promoting EV cargos). This approach remains largely experimental and has not yet reached clinical testing in AD. The second paradigm, which constitutes the majority of current literature, uses EVs as engineered delivery vehicles, where the producer-cell source (commonly MSCs or immune cells) is selected primarily for manufacturability, low immunogenicity, scalability, or intrinsic therapeutic properties, rather than because it recapitulates an endogenous organ–brain signaling axis [160]. The following discussion focuses on this second, more mature paradigm, while explicitly noting where findings might eventually inform the first.

As delivery vehicles, EVs have been used to transport a broad range of payload classes in AD-related models. Small molecules and protein-modulating cargos have included CB2 receptor agonists, quercetin, curcumin, and SHP2-related constructs, with reported effects on neuroinflammation, tau hyperphosphorylation, mitochondrial dysfunction, and cognitive phenotypes [161–163]. Enzyme- or neurotransmitter-related payloads, such as catalase or dopamine, have also been explored as proof-of-concept strategies for reducing oxidative stress or restoring neurotransmitter-associated function; however, much of this work derives from adjacent neurodegenerative fields (e.g., Parkinson’s disease) and should be regarded as cross-disease platform evidence rather than AD-specific validation [164–168].

Nucleic-acid delivery represents one of the most actively pursued EV-based therapeutic directions. Engineered EVs carrying BACE1-targeting siRNA have shown in vivo target suppression and reduced Aβ-related pathology, providing an early proof-of-concept for gene-silencing approaches in the brain [169]. Additional studies report therapeutic benefit using EVs enriched in miR-29 or miR-22 family members, with effects on amyloidogenic processing, inflammatory signaling, and neurological function in AD models [170, 171]. By contrast, examples such as FTO-targeting siRNA delivery by MSC-derived EVs derive from Parkinson’s disease models and should therefore be described as cross-disease platform evidence rather than AD-specific validation [172]. This distinction is important to avoid overstating the maturity of the AD-focused therapeutic literature.

EV source also matters biologically and translationally. While MSC-derived EVs remain the dominant platform in AD-focused studies, EVs from dendritic cells, macrophages, neutrophils, and other immune-cell sources have been explored because of their distinct trafficking properties and immunological interfaces [167, 169, 173, 174]. These sources may offer advantages in some contexts, such as immune modulation or delivery efficiency, but may also introduce additional variability and safety considerations. Likewise, urinary-derived EVs and other scalable biofluid-associated EV sources have attracted interest as accessible engineering substrates. For example, engineered urinary EVs loaded with resveratrol and Pt-based nanozymes and modified with RVG-related targeting strategies have been proposed as multifunctional therapeutic systems in AD models [167]. Such approaches are promising, but their complexity means they should be interpreted as advanced engineering demonstrations rather than near-clinical products. Critically, none of these engineered systems should be conflated with evidence for endogenous pathogenic EV signaling along specific organ–brain axes; they represent a separate, complementary translational effort.

Importantly, EVs may also act as bioactive therapeutics independent of exogenous drug loading. Native or conditioned MSC-derived EVs can carry anti-inflammatory, neurotrophic, or proteostasis-related factors that may themselves contribute to therapeutic benefit. In this sense, the distinction between “carrier” and “cargo” becomes blurred, and EVs may function as both delivery systems and biologically active products. This duality is conceptually attractive, but it complicates dose definition, mechanism-of-action analysis, and product standardization.

Major translational barriers remain. These include standardized production and purification, storage stability, scalable manufacturing, loading efficiency, controlled cargo release, batch-to-batch variability, route-dependent biodistribution, repeated-dosing safety, and the challenge of demonstrating meaningful target engagement in vivo [175] (Table 4).

**Table 4:** Specific mechanisms of EVs as therapeutic vehicles in AD

Key molecule	Disease context	Specific mechanism / strategy	Target	Evidence maturity	Ref
CB2 receptor agonist AM1241	AD model	Encapsulating small-molecule drugs for EV-mediated delivery to enhance brain bioavailability	Demonstrates enhanced brain bioavailability and therapeutic efficacy when delivered via MSC-EVs	Mechanistically informative but not clinically established	[163]
Quercetin, Curcumin, SHP2	AD model	Encapsulating bioactive compounds and protein regulators to counteract AD-related pathologies	Provides therapeutic impetus to counteract hyperphosphorylated tau and mitochondrial dysfunction	Mechanistically informative but not clinically established	[164–166]
Catalase, Dopamine	Non-AD neurodegeneration model (PD)	Encapsulating enzymes and neurotransmitter precursors to alleviate neuroinflammation and replenish neurotransmitters	Alleviates neuroinflammation and replenishes neurotransmitter levels, respectively	Mechanistically informative but not clinically established	[167, 168]
BACE1-targeting siRNA	AD model	Engineering EVs to load siRNA for gene silencing to inhibit pathogenic protein expression	Effectively inhibits BACE1 expression in vivo, thereby suppressing Aβ synthesis	Needs external validation	[169, 176]
miR-29, miR-22 variants	AD model	Utilizing miRNA-enriched EVs to exert neuroprotective effects by modulating gene expression	Downregulates pathological Aβ production and inflammatory cascades in AD models	Mechanistically informative but not clinically established	[170, 171]
FTO-targeting siRNA	Non-AD neurodegeneration model (PD)	Engineering EVs to deliver epigenetic regulators	Delivery via MSC-EVs shows neuroprotective outcomes in a Parkinson’s disease model (mechanism potentially applicable to AD)	Mechanistically informative but not clinically established	[172]
EVs from dendritic cells, macrophages, neutrophils	Platform proof-of-concept	Utilizing EVs from immune cells	DC-EVs can orchestrate immune responses and antigen presentation; certain EVs can evade the reticuloendothelial system, improving delivery efficiency	Exploratory / mechanistically informative	[169, 174]
Resveratrol, Pt nanoparticles	AD model	Luminal loading engineering of EVs for multifunctional therapy	Loaded via methods like electroporation or co-incubation, enabling synergistic antioxidant/catalytic/photothermal functions and targeted delivery	Exploratory / mechanistically informative	[175]
RVG peptide, Transferrin receptor antibodies	Non-AD neurodegeneration model (PD)	Surface engineering of EVs to confer active targeting capability	Precisely directs EVs to target cells like neurons or microglia	Exploratory	[177]

Therefore, while EV-based therapeutic systems represent a highly versatile platform, their development should proceed with careful attention to manufacturability, mechanism, safety, and disease relevance rather than relying primarily on proof-of-concept efficacy in isolated models.

### Targeting EV biology and engineering EVs for disease modification in AD

Beyond using EVs as passive carriers, a broader therapeutic concept is emerging in which EV biology itself becomes a targetable layer of disease modification in AD. This framework is motivated by the possibility that maladaptive EV-mediated communication-particularly from peripheral organs such as the liver, gut, or adipose tissue, may contribute to neuroinflammation, metabolic dysregulation, and BBB vulnerability. In principle, interrupting pathogenic EV release, cargo loading, trafficking, uptake, or downstream signaling could complement conventional CNS-directed therapies by targeting upstream systemic drivers [178, 179]. At present, however, this concept remains more developed at the theoretical and engineering level than at the level of validated disease-modifying interventions in AD (Fig. 5). This line of research directly extends the organ–axis framework from Sect. "Bidirectional communication of EVs in the brain and other organs", but it should be clearly distinguished from the engineered delivery platforms discussed in Sect. "EVs as therapeutic carriers and bioactive therapeutic products", as the two have different mechanistic rationales, target selections, and translational roadmaps.

One branch of this strategy focuses on engineering EVs for more precise CNS delivery and pathway-selective intervention. Surface modification approaches, including RVG-based ligands or transferrin receptor-targeting strategies, have been used to increase EV affinity for neurons, endothelial cells, and microglia [177, 180]. In parallel, EVs can be loaded with siRNAs, miRNA antagonists, neurotrophic regulators, or small molecules through electroporation, sonication, incubation, or producer-cell engineering [181, 182]. Landmark proof-of-concept work using Lamp2b-RVG fusion constructs demonstrated that systemically delivered engineered EVs could preferentially accumulate in neuronal tissues and support CNS nucleic-acid delivery [169]. Subsequent studies extended this paradigm in AD-related settings: MSC-derived RVG-modified EVs have been reported to reduce inflammatory signals and improve cognitive performance in AD models, while engineered EVs carrying a miR-206-3p antagomir have been shown to increase BDNF signaling and ameliorate disease-related phenotypes [183, 184]. These studies support platform feasibility, but should not be conflated with evidence that endogenous pathogenic EV pathways have already been successfully neutralized in AD (Fig. 5).

A second branch aims to alter EV function indirectly by manipulating donor-cell state. Preconditioning MSCs in neurogenesis-supporting environments, culturing them under hypoxic conditions, or modifying bioprocess parameters such as 3D dynamic bioreactor growth can change EV yield, cargo composition, and apparent therapeutic potency [185–187] (Table 5). These strategies are highly relevant for product optimization and manufacturing science. However, they should be framed as therapeutic engineering and production-control approaches rather than as direct evidence for systemic disease-modifying action in patients.

**Table 5:** Representative EV engineering and EV-biology-targeting strategies for disease modification in AD

Key molecule	Specific mechanism / Strategy	Target	Ref
Lamp2b-RVG chimeric protein	Achieving brain targeting via surface modification, enhancing EV accumulation in affected brain regions	Endows EVs with enhanced affinity for neuronal tissue, leading to accumulation in the cortex and hippocampus	[169]
MSC-RVG-EVs	Engineering therapeutic EVs for multi-target synergistic therapy	Not only homes to neural tissue but also downregulates pro-inflammatory cytokines, upregulates IL-10, and improves cognitive function	[183]
MSC-EVs loaded with miR-206-3p antagomir	Engineering EVs to deliver miRNA antagonists, modulating key neurotrophic pathways	Upregulates BDNF, activates the BDNF/TrkB pathway, and thereby ameliorates cognitive deficits	[184]
Culturing AD-MSCs in neurogenesis-conducive or hypoxic conditions	Modulating parent cell culture conditions to custom-produce EVs with specific functions	Produces EVs with enhanced neurogenic potential or enriched with anti-inflammatory miRNAs (e.g., miR-511-3p)	[185, 186]
Culturing hMSCs in a 3D dynamic bioreactor	Scaling up therapeutic EV production using bioreactor technology	Significantly increases EV yield and optimizes their therapeutic cargo profile	[187]

A third and still underdeveloped direction is the selective inhibition of maladaptive EV biology itself. Conceptually, this could include suppressing EV release from pathogenic peripheral sources, blocking brain-relevant uptake of disease-promoting EV populations, or selectively altering harmful cargos while sparing beneficial EV communication. Yet this remains difficult in practice because EV pathways are shared across normal physiology and pathology, source attribution is often incomplete, and the same broad EV class may contain both beneficial and harmful subpopulations. Notably, this third direction is the one most directly aligned with the organ–brain axes discussed in Sect. "Bidirectional communication of EVs in the brain and other organs", but it is also the least mature experimentally. For this reason, disease-modifying strategies targeting EV biology will require far greater source resolution and mechanistic selectivity than is currently available.

Taken together, EV-focused disease modification in AD should be understood as comprising two related but distinct goals: first, engineering EVs to deliver therapeutic signals with improved precision; and second, eventually learning how to suppress maladaptive endogenous EV communication without disrupting physiological intercellular exchange. The field has made substantial progress on the first goal, whereas the second remains conceptually compelling but experimentally immature. Future work should therefore prioritize mechanistic specificity, biodistribution quantification, and manufacturable design over broad claims of system-level disease modification.

## Conclusion and future perspectives

Alzheimer’s disease, long conceptualized as a brain-centered disorder, is increasingly being interpreted within a broader systemic framework in which bidirectional communication between the CNS and peripheral organs may shape disease susceptibility, progression, and therapeutic responsiveness. In this Review, EVs are discussed as one plausible and potentially unifying signaling layer within this inter-organ network. Rather than replacing established mechanisms in metabolism, immunity, vascular biology, or barrier dysfunction, EVs may help explain how these peripheral states are encoded, transported, and translated into brain-relevant consequences.

Three synthesis points emerge from the current literature. First, not all organ axes are supported equally. Among the axes surveyed, the gut–brain axis, including microbiota-derived bacterial EVs [188], together with the metabolically coupled liver–brain and adipose–brain axes, currently shows the strongest convergent support from biological plausibility, disease association, and preclinical mechanistic work [189]. By contrast, the heart–brain, lung–brain, and bone–brain axes remain compelling in selected experimental settings but are still more model-dependent and less consistently supported as endogenous, quantitatively relevant EV pathways in AD.

Second, the major shared limitation across the field is not the lack of candidate cargos, but the lack of source-resolved, quantitative, and causal in vivo evidence. It remains difficult to assign organ-of-origin with high specificity in complex biofluids, to determine how much endogenous EV material actually reaches relevant neurovascular or neural interfaces, to identify recipient cell types with confidence, and to separate EV-specific effects from co-occurring soluble mediators, lipoproteins, protein aggregates, and generalized inflammatory states. As a result, many currently proposed mechanisms remain biologically plausible but not yet decisively established. Building on these synthesis points, a vascular-centric integrative model is proposed in which the cerebral microvascular endothelium and neurovascular unit serve as the shared convergence point for diverse peripheral EV signals. Regardless of organ origin, many EV populations activate common downstream mechanisms like endothelial activation, tight-junction disruption, NF-κB/NLRP3 inflammatory cascades, and impaired perivascular clearance that collectively shape brain vulnerability. This framework provides mechanistic coherence across organ axes while preserving the distinct evidentiary maturity of each axis.

Third, the most actionable next steps are methodological as much as biological. Future progress will likely depend on study designs that combine rigorous EV characterization with clinically meaningful endpoints. Particularly valuable directions include standardized multicenter longitudinal cohorts linking organ-enriched EV measurements to imaging and cognition, source-resolved in vivo tracing strategies, recipient-cell target engagement assays under defined barrier conditions, and perturbation-based experiments that selectively alter EV release, uptake, or defined cargos while controlling for non-vesicular confounders. To make the recommendation for "source-resolved in vivo tracing" more actionable, specific emerging methodologies that are technically feasible are highlighted: (i) Cre-LoxP-based EV reporter systems that enable tissue-specific labeling of EVs and quantification of their biodistribution [190] (e.g., using floxed CD63-GFP or CD81-GFP under a tissue-specific promoter); (ii) bioorthogonal labeling with click chemistry, which allows metabolic labeling of EVs with azide- or alkyne-tagged sugars or lipids followed by in vivo tracking [191]; and (iii) proximity-dependent biotinylation approaches (e.g., TurboID-based labeling of EV interactomes) [192] that can capture cell-type-specific EV release and target engagement. While still primarily in preclinical development, these techniques offer a pathway toward definitive source attribution and causality testing in AD. In parallel, biomarker development will require reproducible pre-analytical pipelines and external validation, whereas therapeutic translation will depend on scalable manufacturing, safety profiling, and robust biodistribution assessment.

Overall, a systemic EV framework broadens the conceptual and translational landscape of AD research, but this broader view must remain disciplined. Progress will come less from expanding lists of candidate cargos and more from establishing origin, dose, destination, mechanism, and clinical relevance. If these challenges can be addressed, EV biology may contribute meaningfully to the development of more reproducible biomarkers and more genuinely disease modifying intervention strategies in AD.

## Data Availability

No datasets were generated or analysed during the current study.

## Abbreviations

AD: Alzheimer’s disease
Aβ: Amyloid-β
AD-MSCs: Adipose-derived mesenchymal stem cells
ALIX: ALG-2-interacting protein X
ANP: Atrial natriuretic peptide
APLP1: Amyloid precursor-like protein 1
apoE: Apolipoprotein E
ATP1A3: ATPase Na+/K+ transporting subunit alpha 3
BBB: Blood–Brain barrier
BDEVs: Brain-derived extracellular vesicles
BDNF: Brain-derived neurotrophic factor
BM-MSCs: Bone marrow mesenchymal stem cells
bEVs: Bacterial extracellular vesicles
CB2: Cannabinoid receptor 2
CKD: Chronic kidney disease
CNS: Central nervous system
CREB: cAMP response element-binding protein
E-selectin: Endothelial selectin
ESCRT: Endosomal sorting complex required for transport
EVs: Extracellular vesicles
ICAM-1: Intercellular adhesion molecule-1
IRS-1: Insulin receptor substrate-1
LPS: Lipopolysaccharide
LRP1: Low-density lipoprotein receptor-related protein 1
MVBs: Multivesicular bodies
NEP: Neprilysin
NF-κB: Nuclear factor kappa B
NLRP3: NOD-like receptor family pyrin domain containing 3
p-tau: Phosphorylated tau
RVG: Rabies virus glycoprotein
SERS: Surface-enhanced Raman scattering
TLR4: Toll-like receptor 4
VCAM-1: Vascular cell adhesion molecule-1
